# Morphological phenotyping after mouse whole embryo culture

**DOI:** 10.3389/fcell.2023.1223849

**Published:** 2023-08-03

**Authors:** Andrew J. Copp, Maryam Clark, Nicholas D. E. Greene

**Affiliations:** Developmental Biology and Cancer, UCL Great Ormond Street Institute of Child Health, London, United Kingdom

**Keywords:** mouse, embryo, culture, neural tube, neurulation, phenotype

## Abstract

Morphological phenotyping of the mouse embryo is described at neurulation stages, primarily as a guide to evaluating the outcome of whole embryo cultures between embryonic days 8.5 and 9.5. During this period, neural tube closure is initiated and progresses to completion in the cranial region. Spinal closure is still underway at the end of the culture period. The focus of this article is particularly on phenotyping that can be performed at the bench, using a stereomicroscope. This involves assessment of embryonic health, through observation and scoring of yolk sac blood circulation, measurement of developmental stage by somite counting, and determination of crown-rump length as a measure of growth. Axial rotation (“turning”) can also be assessed using a simple scoring system. Neural tube closure assessment includes: 1) determining whether closure has been initiated at the Closure 1 site; 2) evaluating the complex steps of cranial neurulation including initiation at Closure sites 2 and 3, and completion of closure at the anterior and hindbrain neuropores; 3) assessment of spinal closure by measurement of posterior neuropore length. Interpretation of defects in neural tube closure requires an appreciation of, first, the stages that particular events are expected to be completed and, second, the correspondence between embryonic landmarks, for example, somite position, and the resulting adult axial levels. Detailed embryonic phenotyping, as described in this article, when combined with the versatile method of whole embryo culture, can form the basis for a wide range of experimental studies in early mouse neural development.

## 1 Introduction

This article summarises some technical and interpretational aspects of morphological phenotyping of mouse embryos at neurulation stages. The focus is particularly on analysis of embryos cultured intact for 24 h from embryonic day E 8.5, before the onset of neural tube closure, to E9.5 when cranial closure is complete. While most detail is given to neural tube phenotyping, other aspects of embryonic development during this period are also covered. We focus particularly on phenotyping that can be performed at the bench, using only a stereomicroscope. Whole embryo culture (WEC) is a valuable tool in mammalian developmental biology (New, 1978; Culshaw et al., 2019; Aguilera-Castrejon et al., 2021) that enables longitudinal analysis of developing mammalian embryos outside the uterine environment, thereby overcoming the limitations of viviparity for specific periods of embryogenesis. Table 1 lists some advantages and limitations of the WEC method, as regards availability and accessibility for study, the role of the maternal environment and aspects of experimental design.

**TABLE 1:** Advantages and limitations of *in vivo* studies versus whole embryo culture (WEC) in mouse and rat developmental biology.

Aspect	*In vivo* studies	Whole embryo culture
Stage availability	Stages from E10.5 onwards are available, whereas earlier stages largely unavailable for direct access due to small size within uterus	Restricted to the period when WEC can be performed: e.g., E7.5-E11.5 as intact embryos Buckley et al. (1978); Aguilera-Castrejon et al. (2021), and E10.5-E12.5 as ‘open yolk sac’ cultures Martin and Cockroft (2008)
Period of follow-up	Can extend to birth, and beyond if pups are delivered by caesarean section and fostered	Up to 4 days culture has been described (see above), with 24 h often providing an adequate developmental period for experimental studies *in vitro*
Embryo accessibility for substance administration	Readily available, via oral, subcutaneous, intravenous or intraperitoneal dosing of the dam. Direct administration to embryo requires maternal surgery	Readily accessible via addition of substances to the culture medium, intra-amniotic injection or cannulation of yolk sac vessels Mensah-Brown et al. (1989)
Embryo accessibility for surgical manipulation	Requires often complex surgical methods to gain access, with general anaesthesia of pregnant dam	Readily accessible throughout the culture period, although may require opening of the yolk sac +/- amnion to gain surgical access to embryo
Maternal metabolism	A factor that must be considered in all *in vivo* studies	Maternal metabolism is removed, which can be used to identify embryo-specific effects, but may fail to identify teratogenic agents that require maternal metabolic activation Ozolins et al. (1995)
Experimental design	The pregnant dam/litter is the unit of experimental design	The individual embryo is the unit of experimental design, as embryos from a single litter can be assigned to different treatment groups, thereby reducing number of pregnant dams required
3Rs considerations	Requires dosing or anaesthesia +/- surgery of pregnant dams	Avoids dosing, anaesthesia or surgery of pregnant dams, although they are euthanised to obtain embryos

## 2 Explanting embryos from the uterus and whole embryo culture

**FIGURE 1:**
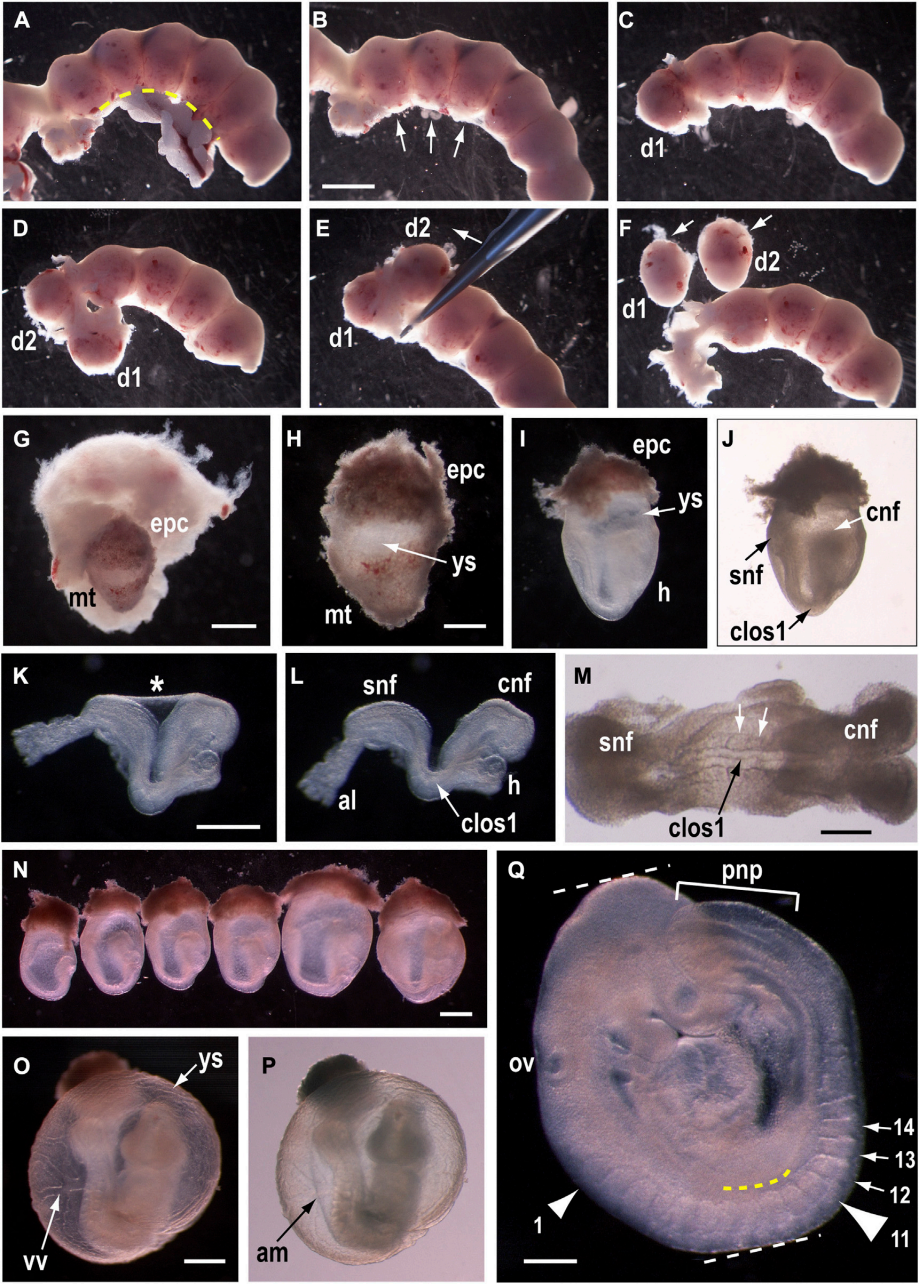
Dissection and culture of mouse embryos from E8.5 to E9.5. **(A–J)** Explanting E8.5 embryos from the uterus. After removal of the uterus from the pregnant female, trim fat and blood vessels away from the mesometrial surface using small scissors [along yellow dashed line in **(A)**]. Subsequent dissection of the uterus should be from the mesometrial surface [arrows in **(B)**]. Using watchmakers forceps, tease apart the muscular uterine wall until the first decidua bulges out [d1 in **(C)**] and continue by exposing each adjacent decidua in turn [d1, d2 in **(D)**]. Clamp forceps around the uterus and gently tease out the decidual swellings [in direction of arrow in **(E)**] until they are free of the uterine wall [d1, d2 in **(F)**]. Dissect into each decidual swelling from its abembryonic pole [arrows in **(F)**], which has a dark red colour and fluffy texture, in contrast to the embryonic pole which is pale coloured and smooth [d1, d2 in **(F)**]. Opening the decidua reveals the embryo encased in its trophoblastic layer **(G)**. Remove the embryo gently from the decidua **(H)**, leaving the future placental trophoblast (epc, ectoplacental cone) intact. Then, using fine watchmakers forceps, remove the mural trophoblast (mt) together with the underlying elastic Reichert’s membrane, as a single layer, tearing around the circumference of the ectoplacental cone. Care must be taken not to damage the yolk sac (ys) which lies directly beneath Reichert’s membrane [arrow in **(H)**]. After removing mural trophoblast and trimming the ectoplacental cone, this 7-somite embryo, encased within its yolk sac and amnion, is ready for WEC **(I, J)**. Note the cranial (cnf) and spinal (snf) neural folds, with the site of Closure 1 (clos1) in between, at the most dorsally concave region of the embryo. The heart **(H)** may have begun to beat. Following culture, or to inspect embryos further, remove the yolk sac and ectoplacental cone. The amnion [asterisk in **(K)**] maintains the embryo in a dorsally concave position, whereas amnion removal allows the embryo to relax and extend rostrocaudally **(L)**. By this stage, the allantois (al) has fused to the chorion, on the underside of the ectoplacental cone, but its attachment is broken by embryo removal. High magnification view of the dorsal embryonic surface reveals completion of Closure 1, as shown by the narrow neural tube profile with midline cavity [black arrow in **(M)**], and flanking somites [white arrows in **(M)**]. **(N–Q)** Whole embryo culture from E8.5 to E9.5. Six embryos from a single E8.5 litter **(N)**, prepared for WEC and viewed from the right side (caudal to left, rostral to right). Embryos are arranged in ascending order of developmental stage, with the least advanced (4 somites, pre-Closure 1) on the left and the most advanced (9 somites, post-Closure 1) on the right. Axial rotation has begun in the 9-somite embryo, as shown by turning of the cranial region towards the viewer, through an angle of ∼45^o^ relative to the body axis. After 24 h culture, embryos have completed axial rotation **(O,P)** and are enclosed in the yolk sac which has a vigorous blood circulation through the vitelline vessels (vv). The amnion (am) can be seen inside the yolk sac, using bright field illumination **(P)**. After removal of the extra-embryonic membranes, embryonic morphology can be scored in detail **(Q)**. Note the completely closed neural tube except for the open PNP (pnp), whose length can be measured (bracketed distance) as an indication of spinal neurulation progress. Somites are counted to determine embryonic stage (e.g., arrows to somites 12–14). The first (small arrowhead) and 11th (large arrowhead) somites are indicated, with the latter located opposite the caudal edge of the forelimb bud (yellow dashed line). Crown-rump length is a linear growth measurement, between the white dashed lines. The otic vesicle (ov) is a landmark that can be used to evaluate the extent of hindbrain closure, which is complete in this 17-somite embryo. Scale bars: 3 mm in B [also **(A)**, **(C–F)**]; 1 mm in G; 0.5 mm in H [also **(I,J)**]; 0.5 mm in K [also **(L)**]; 0.25 mm in M; 0.5 mm in N; 0.5 mm in O (also P); 0.25 mm in Q. Embryos are on a CBA/Ca x C57BL/6 F1 genetic background. Photomicrographs taken on a Zeiss SV11 stereomicroscope, with transmitted light [bright field in **(J,M,P)**; dark field in other images].

Whether embryos are to be cultured or not, most studies of mid-gestation mouse development begin with explanting embryos from the pregnant uterus. Figure 1 shows the main stages of embryo dissection from the uterus (Figure A–J,N), and some aspects of phenotyping at E9.5 after WEC (Figure 1K–M, Figure 1O–Q). We culture mouse embryos by the rolling tube method (New et al., 1973) using rat serum as culture medium, prepared as described (Takahashi et al., 2014). Rat serum can be diluted up to 50:50 in certain culture media without loss of embryo quality (Culshaw et al., 2019).

## 3 General phenotyping following whole mouse embryo culture

**FIGURE 2:**
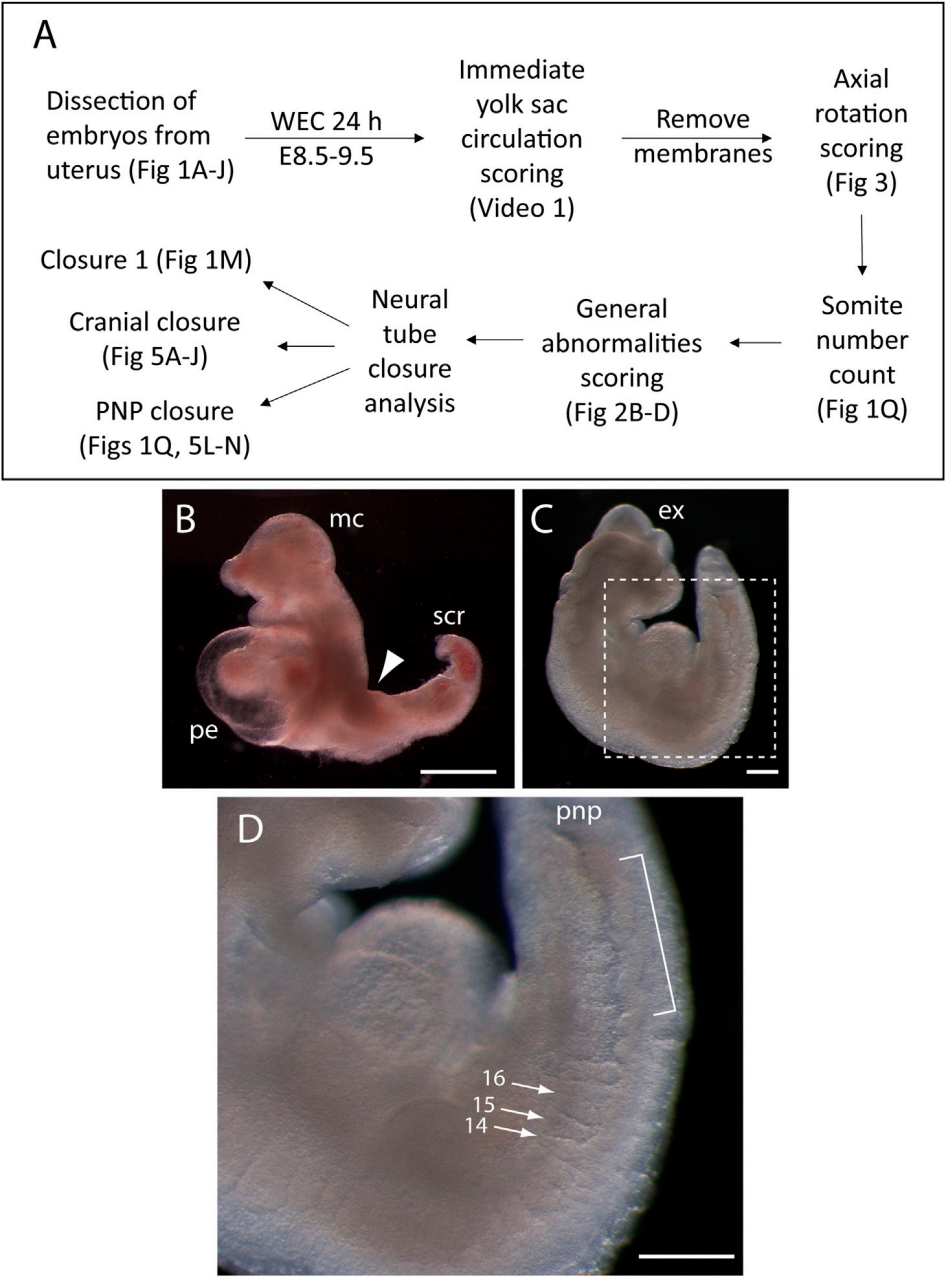
Phenotyping pipeline and developmental abnormalities that may appear in whole embryo cultures for 24 h from E8.5. **(A)** Flow diagram showing the sequential steps of phenotyping embryos after 24 h whole embryo culture (WEC) from E8.5. Relevant figure parts are cited at each step. **(B)** Enlargement of the pericardial sac (pe) in an embryo that is severely growth retarded. Note the lack of axial rotation (arrowhead to dorsal flexure), microcephaly (mc) and stunted caudal region (scr). **(C, D)** Embryo that has almost completed axial rotation, but is growth retarded, with exencephaly (ex) affecting fore-, mid- and hind-brain. Boxed area in **(C)** is shown at higher magnification in **(D)**. Arrows indicate somites 14, 15, and 16 which are irregularly shaped, with the 14th and 15th showing complementary wedge shapes, dorsoventrally, while the 16th is abnormally wide, due to failed separation from the next somite in caudal sequence. Bracketed area: a length of “wavy” neural tube, as seen in dorsolateral view, immediately rostral to the open posterior neuropore (pnp). Scale bars: 0.25 mm.

Figure 2A shows the phenotyping pipeline used in our laboratory with embryos after 24 h WEC from E8.5. The process begins with an initial assessement of embryo health, via scoring of yolk sac circulation and axial rotation, then staging by somite number count, a general survey for typical abnormalities that may occur in sub-optimal cultures, and finally a detailed assessment of the neurulation status of the embryo.

### 3.1 Assessing the health of cultured embryos

Development and maintenance of a functional blood circulation in the yolk sac is a sensitive health index of whole embryo cultures. E8.5 embryos should develop a blood circulation during the first 12 h of culture (Ji et al., 2003), and this should be maintained to the end of the 24 h culture period, and beyond (if studied). At the end of culture, embryos should be scored for yolk sac circulation immediately after removal from the incubator, keeping the embryos within the warm culture serum, as blood circulation diminishes rapidly when embryos cool down (Supplementary Video S1; Supplementary Figure S1). We use a simple scoring system: e.g., +++, vigorous, continuous circulation throughout the yolk sac; ++ extensive but discontinuous circulation that ceases between heart beats; +, patchy circulation in which only a few blood cells are seen to move; -, no circulation. We exclude embryos from analysis if they lack a yolk sac circulation (i.e., score of -), as they are effectively dead, and will very likely exhibit abnormalities.

### 3.2 Abnormalities resulting from yolk sac circulation failure

**FIGURE 3:**
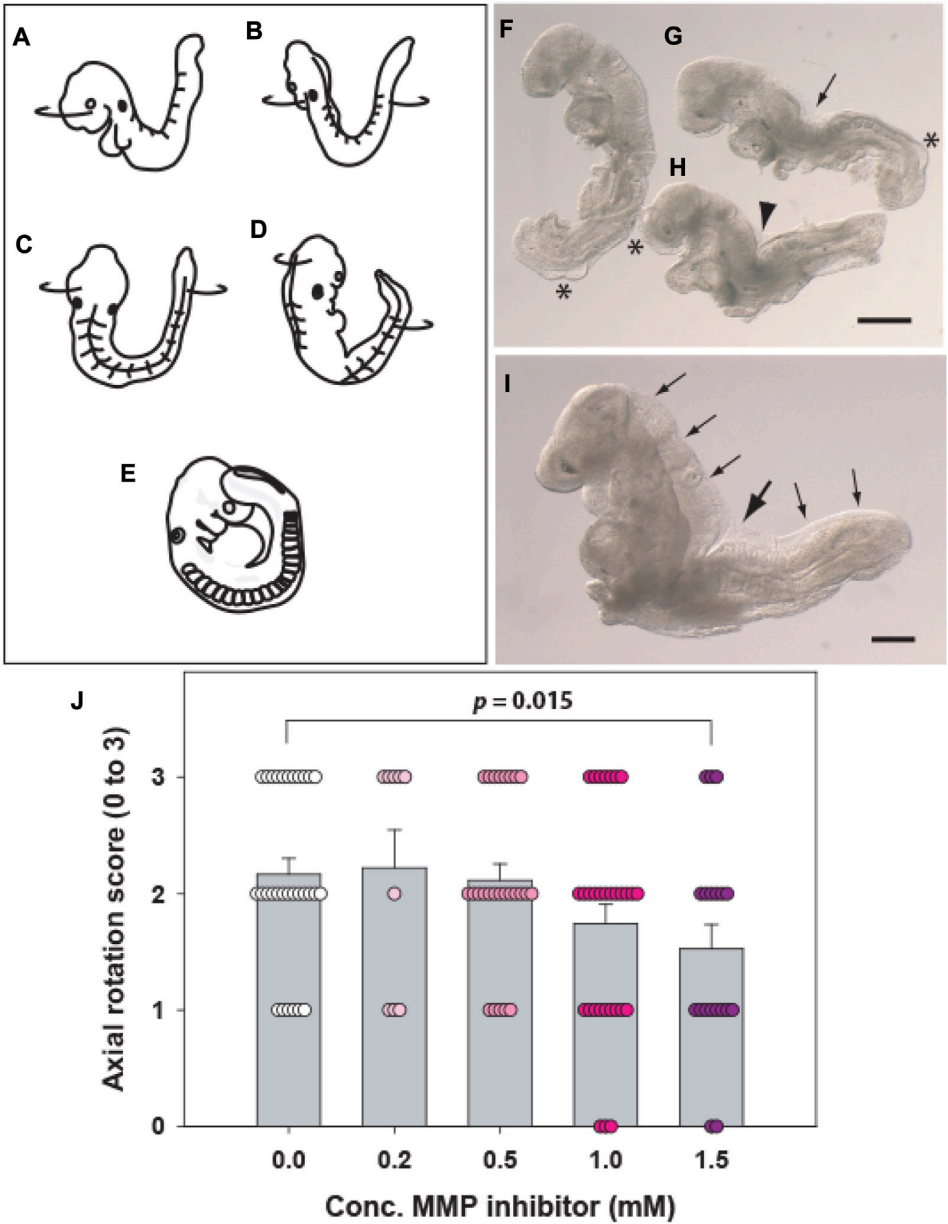
Axial rotation (“turning”). **(A–E)** Diagram showing the axial rotation process, which begins around the 9-somite stage when the embryonic head turns to the right side relative to the upper body axis **(A)**. Soon afterwards, the lower body axis begins to rotate anticlockwise **(B)**, until an intermediate position is reached in which the entire ventral embryonic surface faces the right, with the entire dorsal surface facing the left **(C)**. Rotation of both head and trunk continues **(D)** until the embryo achieves the dorsally convex, fetal position, with its caudal region adjacent to the right side of the head **(E)**. **(F–H)** Embryos with abnormalities of axial rotation following 24 h culture from E8.5. Embryos tend to ‘maximise’ their turning after removal from the yolk sac and amnion, but abnormalities can still be detected as failure of the caudal region to reach the right side of the head (F; ++ score), a straight body axis with persistence of a dorsal indentation (arrow in G; + score) or complete failure of turning with dorsal flexure midway along the body (arrowhead in H; - score). Asterisks: surface blebs indicative of poor culture outcome. **(I)** *Vangl2* ^*Lp/Lp*^ mutant embryo with entirely open neural tube (craniorachischisis, small arrows) and complete lack of turning, with strong dorsal flexure (large arrow). **(J)** Example of axial rotation assessment (3: fully turned; 0: unturned) in a study of matrix metalloproteinase (MMP) inhibitor treatment of wild-type embryos cultured for 24 h from E8.5. A dose-dependent reduction in axial rotation is seen, with 1.5 mM inhibitor differing significantly from vehicle control. Each symbol is a separate embryo. MMP-2/9 inhibitor (CAS Number: 193807–58–8) was dissolved in dimethyl sulfoxide, with addition of 0.1% v/v stock solution to cultures. Diagram **(A–E)** modified with permission from Figure 2 of Deuchar (1975) which depicts axial rotation in the rat embryo. Scale bars: 0.5 mm in **(F–H)**; 0.25 mm in **(I)**.

Nutrient and gas exchange through the yolk sac (termed “histiotrophic” nutrition) underlies growth and development during early mouse organogenesis (Lloyd et al., 1985; Beckman et al., 1990), and enables the WEC approach in which the embryo develops in a liquid culture environment, rather than within the uterine implantation site. This yolk sac-dependent phase precedes the onset of placental function (∼E10.5 in mouse) and hence yolk sac-mediated transport between mother and embryo covers the period when many major birth defects arise (D'Souza et al., 2021). If yolk sac circulation fails prematurely during WEC, embryos exhibit increasing signs of degeneration, with expansion of the fluid-filled cavities, particularly pericardial enlargement (edema, Figure 2B), and excessive swelling of the yolk sac cavity (exocoelom) (Supplementary Video S1). Other features that may result from nutritional deprivation include microcephaly, irregularity of newly formed somites, waviness of the neural tube (Figures 2C,D), hemorrhage and sub-epidermal fluid-filled blebs (Figures 3F–H), particularly on the cranial region. Heartbeat is sometimes used as a sign of embryonic health, but at neurulation stages this can be misleading as the heart’s intrinsically contractile nature means it may continue beating even in moribund embryos (Supplementary Video S1). Healthy embryos should be further assessed as described in Section 3.3, Section 3.4.

### 3.3 Determining embryonic stage–Days post coitum versus somite number

Mice are usually paired overnight for mating, and the finding of a copulation plug is assumed to indicate mating midway through the preceding dark period of the day-night cycle. We designate 13:00 on the day of finding a plug as 0.5 days post-coitum, or embryonic day 0.5 (E0.5), as this is approximately 12 h after mating. Precise staging during the period E8.5 to 13.5 is done by determining somite number, which is a more accurate measure than days post coitum: developmental events tend to occur within a small range of somite stages, irrespective of chronological timing. In contrast, embryos of different strain backgrounds can reach particular somite stages (and hence developmental events) at widely differing ages post coitum. Even within litters, there is usually a range of stages that varies with genetic background, and is partially due to the relatively earlier stage reached by female embryos compared with males (Brook et al., 1994). Generally, outbred strains are more “advanced” than inbred strains, requiring adjustment of experimental timings, depending on the strain that is used. A staging system has been described for pre-somite stage embryos (Downs and Davies, 1993), which is valuable if embryos are to be cultured before E8.5 (e.g., from late gastrulation stage, E7.5).

### 3.4 Determining somite number after embryo culture

Somites can be counted in embryos following culture, once the yolk sac and amnion have been removed (Figure 1Q). When staging E9.5 embryos with fewer than 20 somites, the count can begin with the first somite, which is located in the occipital region, ventro-caudal to the developing otic vesicle. In more advanced embryos, the occipital somites become ill-defined and may be missed giving a falsely low somite count. In this case, the count should begin at somite 11, situated level with the caudal edge of the forelimb bud (Figure 1Q). For embryos with 30 somites or more, the rostral somites may be indistinct and counting can begin with somite 29, located opposite the caudal edge of the hindlimb bud. The yolk sac and amnion must be kept intact for embryo culture, hampering a direct count of somites in embryos before culture. Although somites can be counted through the ventral surface of the embryo, prior to axial rotation, this may be inaccurate as the most rostral and caudal somites are not always visible. We calculate somite number increase (in healthy embryos) by assuming addition of one somite every 2 h of culture. Hence a 17-somite embryo should have added 12 somites during a 24 h culture period, and is estimated to have had 5 somites when placed into culture.

### 3.5 Brown-Fabro morphological score

This scoring system (Brown and Fabro, 1981) provides an overall morphological score for individual rat embryos based on a system-by-system assessment of 17 different aspects of the embryo and its membranes, including yolk sac circulation and somite number. But this broad coverage of body systems means the score is rather insensitive to specific malformations. For example, isolated midbrain exencephaly reduces the morphological score of an embryo at E10.5 by less than 5%, which may not be statistically significant, and therefore may hinder linking a malformation of biological significance with a genetic lesion or embryo treatment. The technique is time-consuming, but is very useful for teaching embryonic anatomy. Variants of this scoring system have been described, as applied to mouse embryos (Van Maele-Fabry et al., 1990) and in rats to facilitate identification of developmental anomalies (Zhang et al., 2010).

### 3.6 Growth measurement

Embryonic mass correlates with increasing embryonic age and somite number (Brown and Fabro, 1981), but growth and developmental advancement can become dissociated under experimental or pathological conditions, as in human “intrauterine growth restriction” (IUGR) (Sharma et al., 2017). The most accurate measure of embryonic growth, for non-mutant embryos and experimental conditions that do not directly target protein turnover, is total protein content, for example, using the bicinchoninic acid assay (Smith et al., 1985) provided by the Pierce™ BCA Protein Assay Kit. Alternatively, the measure of crown-rump length (Figure 1Q) is easily performed in fully-turned embryos using an eyepiece graticule on a stereomicroscope, and is compatible with other subsequent modes of analysis.

### 3.7 Axial rotation

This process, also called “turning,” occurs in mouse embryos between the 9- and 13-somite stages, when the body axis rotates from a dorsally-concave “inverted” conformation to the dorsally-convex “fetal” position (Deuchar, 1975). Rotation begins with turning of the embryonic head to the right (Figure 1N, embryo on far right), and the lower body then rotates anti-clockwise, until the fetal position is achieved (Figures 3A–E). This rotation has the important effect of closing the midgut region, and “wrapping” the yolk sac around the entire embryo. Axial rotation is a feature of early embryonic asymmetry, along with asymmetric heart looping, although ∼5% of wild-type embryos spontaneously rotate in the opposite direction. The process is randomised in the *iv* (situs inversus viscerum) mutant: an allele of the *Dnah11* gene (Supp et al., 1997) and is completely reversed in the *Invs* (inversin) mutant (Mochizuki et al., 1998). As with yolk sac circulation, a simple scoring system can be applied, for example: fully turned, +++ (Figure 3E); incomplete but more than 50% turned, ++ (Figure 3D); less than 50% turned, + (Figures 3B,C); completely unturned, - (Figure 3A). Axial rotation is frequently disturbed in WEC, either by suboptimal culture conditions (Figures 3F–H) or due to co-existing developmental anomalies, such as failure of Closure 1 (Figure 3I). An example of an axial rotation analysis from a study by the authors is shown in Figure 3J.

## 4 Assessing the neurulation status of mouse embryos

**FIGURE 4:**
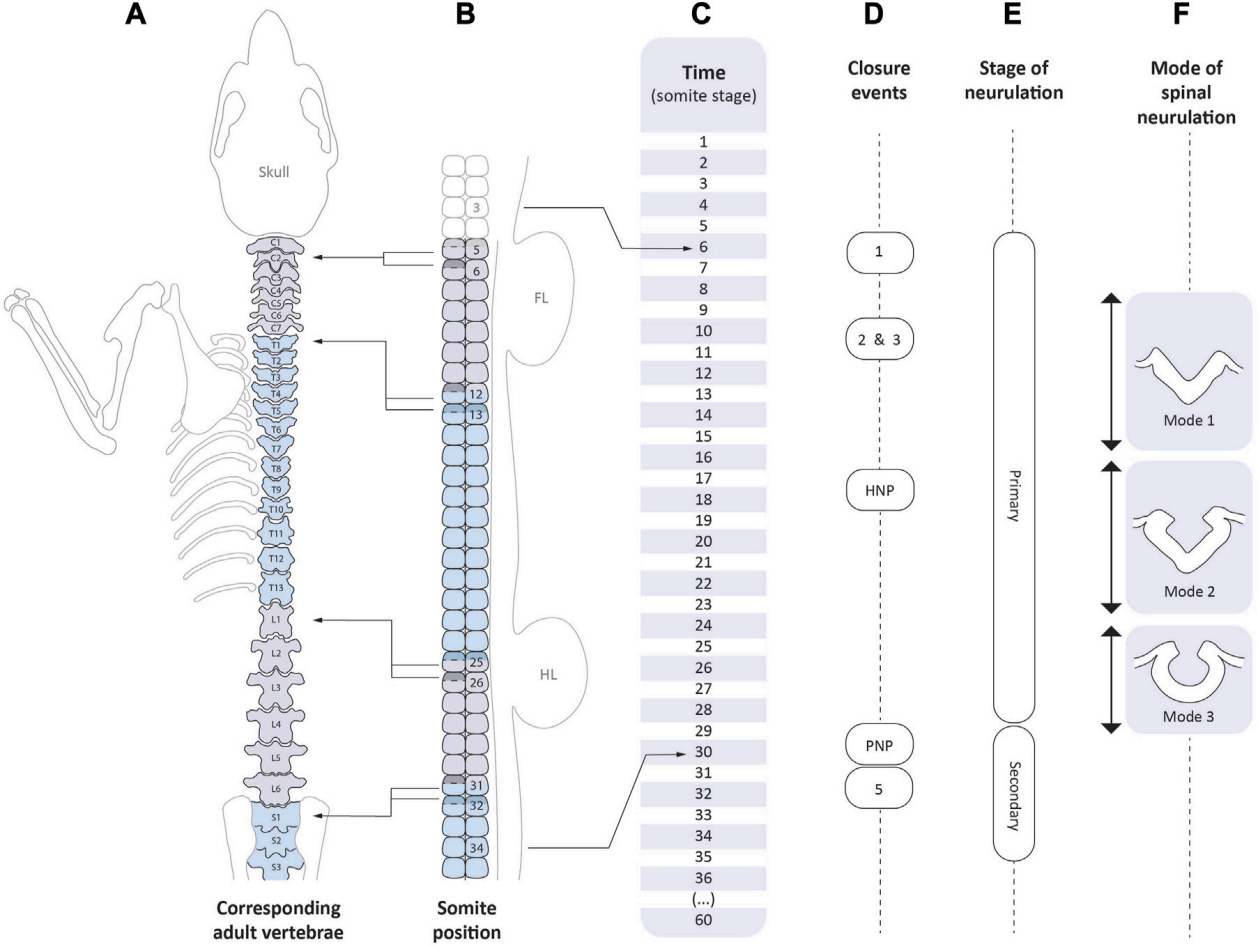
Correspondence between the events of axial development and neurulation. Time-line of mouse development as expressed in somite stages (C, centre). The scale extends from 1 somite (late E7.5/early E8.5), to 36 somites (E10.5), and then to completion of the embryonic period at 60 somites (E13.5). Vertebral levels **(A)** are shown on the left: C1-7, cervical; T1-13, thoracic; L1-6, lumbar; S1-3 upper sacral). Left-pointing arrows indicate the somite positions **(B)** from which certain vertebrae arise. Each vertebral body is formed by the caudal half (unshaded) of one somitic sclerotome and the rostral half (shaded) of the following one; the so-called “resegmentation” process (Christ et al., 1998). Right-pointing arrows indicate the range of somite positions that span mouse primary spinal neurulation **(E)**. Closure events **(D)** begin with Closure 1 which occurs at the level of somite 3 (occipital) in embryos with 6 somites, and ending with PNP closure which occurs at the level of future somite 34 (third sacral segment) in embryos with 30 somites. Hence, the spinal zippering point travels 31 somite-lengths during 24 somite stages, and so the pace of zippering outstrips the rate of somite formation. By the stage of PNP closure, presomitic mesoderm containing at least 4 incipient somites (Copp and Brook, 1989) intervenes between the closing PNP and the most recently formed somite (i.e., number 30), accounting for the final PNP closure position at the 34th somite. Cranial neurulation events: Closures 2 and 3, and hindbrain neuropore (HNP) closure, are shown in relation to the time-line **(C)** only. Modes of spinal neurulation [**(F)**, right side] are indicated in relation to the time-course of primary spinal neurulation. Secondary neurulation **(E)** occurs from the 31 somite stage onwards, until the end of axial development at the 60 somite stage.

Mouse neural tube closure comprises two phases (Figure 4E): primary, in which neural folds bend and close dorsally to create the neural tube (E8.5 to E10.5), and secondary in which the caudal-most neural tube is formed by a mesenchyme-to-epithelium transition in the tailbud, after primary neurulation is complete (E10.5 to E13.5) (Greene and Copp, 2014). Primary neurulation is underway during WEC at E8.5-E9.5. It begins at the six to seven somite stage and finishes at the 29–30 somite stage (Figures 4C,D), therefore taking just less than 2 days to be completed. Neurulation phenotyping involves determining whether and to what extent the neural folds are open, which closure events have occurred successfully, which have failed, whether any abnormalities are the result of developmental defect or dissection artifact, and how far abnormalities at a particular stage are predictive of future developmental defects, termed neural tube defects (NTDs) which are common birth defects in humans (Copp et al., 2015). It is important to bear in mind that neurulation may be disrupted at more than one axial level in a single individual, as seen, for example, in the *Pax3* (*splotch*) mouse mutants that exhibit both cranial and spinal defects (Greene et al., 2009).

### 4.1 The sequence of neurulation events

Primary neurulation is a discontinuous, piecemeal process involving considerable heterogeneity along the body axis. Closure is initiated at three distinct sites (Closures 1–3; Figure 4D) within the brain and upper spinal cord, and neurulation progresses by unilateral or bilateral “zippering” between these sites. Hence, closure can progress in either a rostral or caudal direction, depending on axial level. The open regions of neural plate between the closure points are termed neuropores, and these are the last parts of the neural tube to close in a particular body region. Table 2 shows the main neural tube closure events, and gives suggestions on how best to observe them after WEC.

**TABLE 2:** Assessing neurulation events in the mouse embryo.

Closure event	Description of event	Gestational age^a^	Somite range	Method of assessment
Closure 1	Initiation of closure at occipital/cervical boundary	E8.5	6–7	Gross observation from dorsal view, in embryos removed from yolk sac and amnion. Transection of body axis allows closure to be confirmed in neural tube cross-section (Figure 1M)
Closure 2	Initiation of closure at forebrain/midbrain boundary^b^	Late E8.5 or early E9.5	10–13	Gross observation from anterior view, usually in embryos removed from yolk sac and amnion (Figures 5B, C)
Closure 3	Initiation of closure at the rostral extremity of the neural plate	Late E8.5 or early E9.5	10–13	Gross observation from anterior view, usually in embryos removed from yolk sac and amnion (Figures 5B, C)
Anterior neuropore (ANP) closure	Completes closure in forebrain, by progression from Closures 2 and 3	Late E8.5 or early E9.5	12–15	Gross observation from anterior view, usually in embryos removed from yolk sac and amnion (Figures 5C, D)
Hindbrain neuropore (HNP) closure	Completes closure in hindbrain, by progression from Closures 1 and 2	Early E9.5	16–17	Gross observation from posterior view in embryos removed from yolk sac and amnion. Owing to the thin roof of the 4^th^ ventricle of the hindbrain, it may be necessary to confirm closure by gentle pushing with a blunt probe (Figures 5H, I)
Closure 5	Caudal “lip” of the PNP that appears to zipper rostrally Galea et al. (2017)	Early E10.5	25–30	Gross observation from a dorsolateral oblique view of the caudal region in embryos removed from yolk sac and amnion
Posterior neuropore (PNP) closure	Completes closure in the spinal region, by progression from Closures 1 and 5	Early E10.5	29–30	Gross observation, with or without measurement, from a dorsolateral oblique view of the caudal region in embryos removed from yolk sac and amnion (Figure 1Q)
Secondary neurulation	Formation of the neural tube in the low spinal region, caudal to the posterior neuropore	E10.5 onwards	31–60	Requires physical or virtual section analysis

^a^ Gestational days are given as E8.5, E9.5 and E10.5 (24 h periods starting and ending at 1 am). “Early” and “late” are used to indicate stages that occur in the first half or second half of each gestational day. In some cases (e.g., late E8.5/early 9.5) this may indicate events that occur during the night.

^b^ Closure 2 site varies between inbred strains and can be located within the midbrain or within the forebrain (Fleming and Copp, 2000).

### 4.2 Neural tube closure in the context of the body axis

The caudal region at E9.5 is frequently referred to as the “tail” of the embryo, even though the posterior neuropore (PNP) at the 15 somite stage actually corresponds to the future mid-thoracic level of the fetus and postnatal individual. Abnormalities of neuropore closure at this level therefore affect the thorax, not the extreme caudal end, which forms much later in development. Figure 4 summarises the sequence of neurulation events and shows the correspondence between developmental (somite) stage, somite position, and axial level in terms of position along the future vertebral column. When primary neurulation is completed with final closure of the PNP at the 29–30 somite stage, the site of the closing neuropore corresponds to the level of the future 34^th^ somite, which is within the upper sacral region (Figures 4A–C). Thus open spina bifida, which in humans most often affects the lumbo-sacral level, corresponds to faulty closure of the PNP (Copp and Brook, 1989). In contrast, lower spinal disorders (called “spinal dysraphism”), that affect low sacral and caudal levels, result from abnormalities of secondary neurulation.

### 4.3 Closure 1

Neural tube closure initiates at the hindbrain/cervical boundary in embryos with six to seven somites (Table 2; Figure 4), when the open neural groove closes *de novo* at the level of the third somite (Sakai, 1989). Closure initiates at a similar stage and somite level in human embryos (O'Rahilly and Müller, 2002). Failure of Closure 1 generates the most severe type of NTD, termed “craniorachischisis” (CRN; Figure 3I), in which the neural tube remains open from midbrain to low spine (Copp et al., 1994), a defect that comprises around 10% of human NTDs (Moore et al., 1997). Initiation of neural tube closure requires signalling via the planar cell polarity (PCP) pathway, a non-canonical Wnt-dishevelled cascade, that regulates the process of convergent extension (CE) in which cells intercalate towards the midline, driving medio-lateral narrowing and rostro-caudal elongation. This serves to shape the embryo during and following gastrulation (Skoglund and Keller, 2010). Closure 1 is disrupted in mice homozygous or doubly heterozygous for mutations in the PCP pathway, making this the primary signalling cascade required for initiation of neural tube closure (Wallingford, 2012; Murdoch et al., 2014).

To evaluate embryos for completion of Closure 1, they must have reached at least 8 somites, so they are beyond the stage that Closure 1 is normally completed. Using a stereomicroscope, the embryo should be dissected free of its extraembryonic membranes, including yolk sac and amnion. If axial rotation is incomplete (Figures 3A,H,I), the embryo can be gently flattened with forceps, so the Closure 1 region can be inspected. Usually, by varying the angle of view, it is readily apparent whether or not the neural folds have closed at the level of the third somite. In normal embryos, a stretch of closed neural tube will be visible, flanked by the upper somites (Figure 1M). This transitions via the rostral zippering point into the open hindbrain, and via the caudal zippering point into the PNP. Embryos with failed Closure 1 show parallel neural folds that do not meet in the midline, a lack of zippering points rostrally or caudally, and a neural groove that is continuous along the body axis.

To confirm the gross morphological appearance, the Closure 1 region can be transected at the midline with a sharp blade, and then the cross-sectional cut surfaces are observed on the stereomicroscope or by other means (e.g., confocal microscopy). Histological sections can also be cut transverse to the body axis. In cases of PCP genetic mutation, Closure 1 failure is usually accompanied by a short body axis (faulty CE) and incomplete axial rotation (Figure 3I) (Ybot-Gonzalez et al., 2007b). However, Closure 1 failure can occur with normal axial elongation (Nychyk et al., 2022), so these features are not reliable indicators of faulty Closure 1.

### 4.4 Cranial closure

**FIGURE 5:**
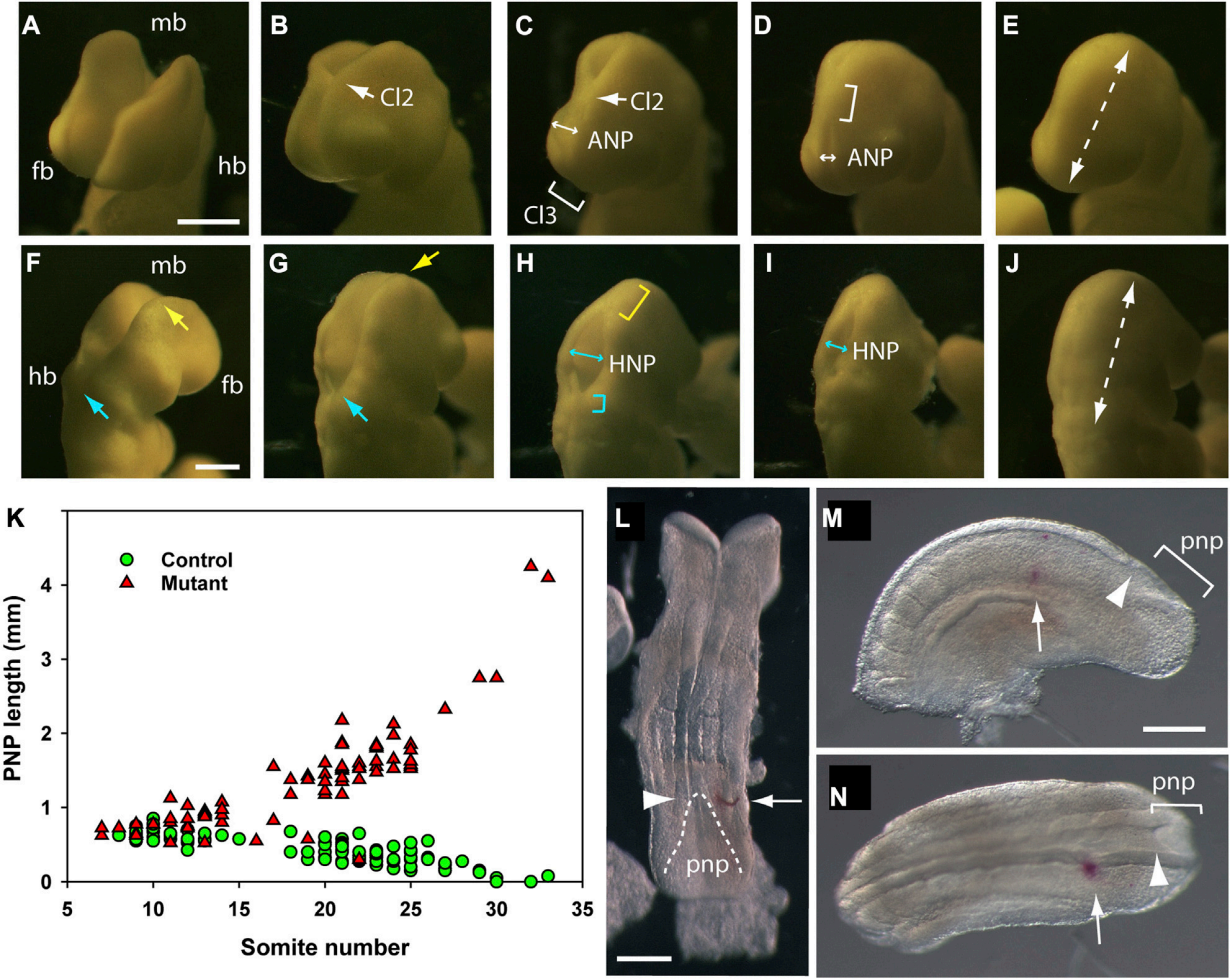
Cranial and spinal closure events, as observed in mouse embryos after 24 h WEC. The developing cranial region of embryos (14–18 somite range) as viewed from the front/left aspect **(A–E)**, to show forebrain (fb) and midbrain (mb) closure and from the back/right aspect **(F–J)**, to show hindbrain (hb) and midbrain closure. **(A–E)** The forebrain and midbrain are initially wide open **(A)**, until the neural folds at the forebrain/midbrain boundary approach each other in the midline to form Closure 2 [Cl2, arrow in **(B)**]. The anterior neuropore (ANP) is a region of open forebrain neural folds between rostrally directed zippering from Closure 2 and caudally directed zippering from Closure 3 [Cl3, bracket in **(C)**]. The ANP narrows rapidly [double headed arrows in **(C,D)**] to complete forebrain closure [dashed arrow in **(E)**]. Zippering from Closure 2 progresses caudally throughout the midbrain [bracket in **(D)**], passes over the apex of the cranial flexure, and then participates in hindbrain closure **(G and H)**. **(F–J)** Hindbrain zippering has progressed in a rostral direction from Closure 1 to the level of the otic vesicle [cyan arrow in **(F)**], while the midbrain neural folds remain wide open [yellow arrow in **(F)**]. Midbrain closure then begins, with caudally directed zippering from Closure 2 [yellow arrow in **(G)**] while hindbrain zippering continues in a rostral direction [cyan arrow in **(G)**]. Once midbrain zippering has passed over the apex of the cranial flexure, it progresses rapidly into the hindbrain [yellow bracket in **(H)**] whereas rostrally directed zippering in the lower hindbrain is less rapid [cyan bracket in **(H)**] (Maniou et al., 2021). These two zippers define the intervening hindbrain neuropore (HNP) which at this stage is relatively wide [double headed arrow in **(H)**], but narrows and shortens progressively [double headed arrow in **(I)**] until the entirely hindbrain is closed [dashed arrow in **(J)**]. This is the last event of cranial neurulation. Photomicrographs of ten different embryos on a CBA/Ca x C57BL/6 F1 genetic background after 24 h WEC from E8.5. Following fixation in Bouin’s fluid, embryos were temporarily mounted in wells of an agarose plate, to enable precise orientation, with photography using incident illumination, directed horizontally from the left side. **(K)** Posterior neuropore (PNP) length in non-cultured embryos of different somite stages. Data reproduced from Figure 3B of De Castro et al. (2018). Controls (green symbols: wild-type or *Grhl3*-floxed) show progressive PNP shortening until complete closure occurs at the 30-somite stage, whereas mutants (red symbols: *Grhl3*-null) show increasing PNP length from the 15-somite stage, preceding development of open spina bifida. **(L–N)** DiI marking of the PNP zippering point to calculate rate of spinal closure. **(L)** Late E8.5 embryo dissected from its membranes immediately after DiI was injected beneath the surface ectoderm (arrow), level with the zippering point (arrowhead). Dashed line outlines PNP neural folds. **(M,N)** Caudal part of E9.5 embryo [**(M)**: left lateral view; N: dorsal view] that was injected with DiI to mark the zippering point and then cultured for 6 h. The DiI mark (arrows) is now separated from the more caudally located zippering point (arrowheads) by approximately 3 somite lengths. Distance between DiI mark and zippering point, divided by time in culture, gives the rate of zippering. Scale bars: 0.25 mm in A [also **(B–E)**]; 0.25 mm in F [also **G–J)**]; 0.25 mm in L; 0.25 mm in M [also **(N)**].

Closure of the future brain is complex, involving several distinct events that may succeed or fail, sometimes independently of each other (Figures 4, 5; Table 2). Closure 2 occurs at the forebrain-midbrain boundary in most strains (Juriloff et al., 1991), with first contact of the apposing neural folds occurring from the 12-somite stage onwards (Figures 5A–C). Closure 3 begins from the rostral extremity of the neural plate, around the same stage as Closure 2 and zippers in a caudal direction (Figure 5C), thereby defining the anterior (rostral) neuropore (ANP) as a region of open neural folds between Closures 2 and 3 (Figures 5C,D). The ANP closes bidirectionally, and is sealed completely in most embryos by the 16-somite stage (Figure 5E). Simultaneously, zippering occurs in a caudal direction from Closure 2, narrowing and eventually closing the midbrain region (i.e., over the apex of the embryonic head; Figures 5F–H). Zippering also travels in a rostral direction from Closure 1 (Figures 5G,H), hence delineating a hindbrain neuropore (HNP), which narrows and closes bidirectionally (Figures 5H,I), with completion of closure by the 16–18 somite stage (Figure 5J), marking the end of cranial neural tube closure.

Embryo culture studies in the literature typically score the embryonic brain as either normal or “exencephalic,” with the latter referring to persistently open cranial neural folds, beyond the somite stage that these should be closed. This is an over-simplification, however, and risks missing more subtle or region-specific anomalies of cranial closure. For example, the forebrain may close according to a normal time-schedule, but hindbrain closure may be specifically defective, as in embryos lacking the Jnk1/2 protein kinases (Kuan et al., 1999), or conversely the forebrain may remain open (often called “split face”), as in *Hes1* gene knockouts (Ishibashi et al., 1995). Most commonly, the midbrain neural folds remain open. Hence, subjecting E9.5 embryos to a more detailed cranial closure analysis (Table 2; Figure 5) allows the investigator to capture information on each of the events of cranial neurulation.

### 4.5 Spinal closure

The spinal region closes by a caudally-directed zippering that begins with Closure 1 at the six to eight somite stage and ends with PNP closure at the 29–30 somite stage (Figures 4C–E). Failure of closure to be completed leads to open spina bifida, while delayed closure often causes a “curled tail” phenotype (Copp, 1985). Live imaging of spinal zippering during WEC has been achieved over relatively short time-frames (Mole et al., 2023). Although the zippering process appears continuous along the spinal region, it is heterogeneous in several respects. First, the closing neural folds are initially flanked by epithelial somites (between Closure 1 and ∼10 somites), but after this stage the zippering point progression “outstrips” the formation of new somites, and becomes flanked by unsegmented presomitic mesoderm. Second, there are three successive phases of spinal neurulation, modes one to three, in which the closing neural folds exhibit different morphology (Figure 4F). In mode 1 the neural plate bends only at the midline, at the median hinge point (MHP). By contrast, in mode 2, the neural plate also shows dorsolateral hinge points (DLHPs) in addition to the MHP, and in mode 3 the MHP is no longer evident, and PNP closure is completed by bending at DLHPs only (Shum and Copp, 1996). Dorsoventral signalling, involving sonic hedgehog (Shh) and bone morphogenetic proteins (BMPs), regulates this changing closure morphology along the spinal axis (Ybot-Gonzalez et al., 2002; Ybot-Gonzalez et al., 2007a). Third, there is a gradual shortening of the PNP region as spinal neurulation occurs, indicating that the rate of neural tube zippering along the embryonic body is slightly more rapid that longitudinal growth of the body axis (Van Straaten et al., 1992). Towards the end of spinal closure, PNP shortening becomes particularly rapid, as a result of a short-lived Closure 5 site which arises at the extreme caudal end of the embryonic axis (Galea et al., 2017). Hence, final sealing of the neuropore is by bidirectional zippering, in a caudal direction from Closure 1 and in a rostral direction from Closure 5. While this final phase of PNP closure is occurring, secondary neurulation is already underway in the tailbud (Shum and Copp, 1996), with direct continuity of the primary and secondary neural tube lumens.

Quantitative assessment of spinal neurulation after WEC involves measurement of PNP length, as a linear distance between the zippering point and the caudal end of the embryo (Figure 1Q). A graph of PNP length against somite number, involving multiple embryos at different somite stages, can be used to determine whether spinal closure is normal or abnormal, for example, when comparing drug-treated versus vehicle-treated control embryos, or mutant versus wild-type embryos (Figure 5K). To gain information on PNP closure rate in individual embryos, we mark the axial level of the zippering point before culture, by creating a small window in the yolk sac, and using a fine glass micropipette to penetrate the intact amnion, and inject a spot of the lipophilic dye DiI beneath the lateral surface ectoderm (Figure 5L). Then, following a period of WEC, the distance travelled by the zippering point beyond the DiI mark (Figure 5M,N), divided by the culture period in hours, provides the rate of spinal zippering for that embryo. This method was used to demonstrate partial rescue of PNP closure by the myosin II inhibitor, Blebbistatin, in *Axd* mutant embryos that over-express the *Grhl2* gene (Nikolopoulou et al., 2019).

### 4.6 Interpreting apparently “abnormal” neural tube closure

The finding of an open neural tube at late embryonic (e.g., E10.5-E13.5) or fetal (E14.5-birth) stages should prompt a study of embryos earlier in development to determine whether this defect indeed represents failure of neural tube closure. An alternative mechanism is re-opening of the closed neural tube, which has been described in cyclophosphamide-treated rat embryos (Padmanabhan, 1988). Moreover, it is important to categorise mutants with open neural tubes according to which closure site or neuropore is compromised. This is especially important for cranial closure defects where the complexity of closure events in different brain regions can generate varying phenotypes (see Section 4.4 above).

In some cases, teratogen-treated or genetically mutant embryos die around mid-gestation (e.g., E9.5) and exhibit an open neural tube. Before concluding that an NTD is present, it is important to determine whether such embryos progress far enough in development to have reached the normal (somite) stage of a particular neural tube closure event. For example, although E9.5 embryos homozygous for the *csk* gene knockout exhibit open cranial neural tubes (Imamoto and Soriano, 1993), they are developmentally retarded and appear to die before reaching the stage at which the cranial neural tube would have closed in normal development. Hence, such embryos cannot be safely concluded to have NTDs.

## 5 Conclusion

Culture of intact mouse embryos offers an opportunity to perform a range of experimental studies that are often considered unfeasible in mammals, due to their viviparity. Accurate phenotyping of cultured embryos is important to ensure that the effects of an experimental perturbation, whether genetic or environmental, are correctly identified and interpreted, and that different prospective birth defects can be recognised, despite the early developmental stage at which analysis is performed. With ongoing developments in live imaging, the whole embryo culture method can be expected to yield further insights into mouse embryonic development and the origin of developmental disorders.
