# Supplementary figures and images for: Morphological phenotyping after mouse whole embryo culture
Source: Front Cell Dev Biol. 2023 Aug 3;11:1223849. doi: 10.3389/fcell.2023.1223849 (PMC10435082; doi:10.3389/fcell.2023.1223849)

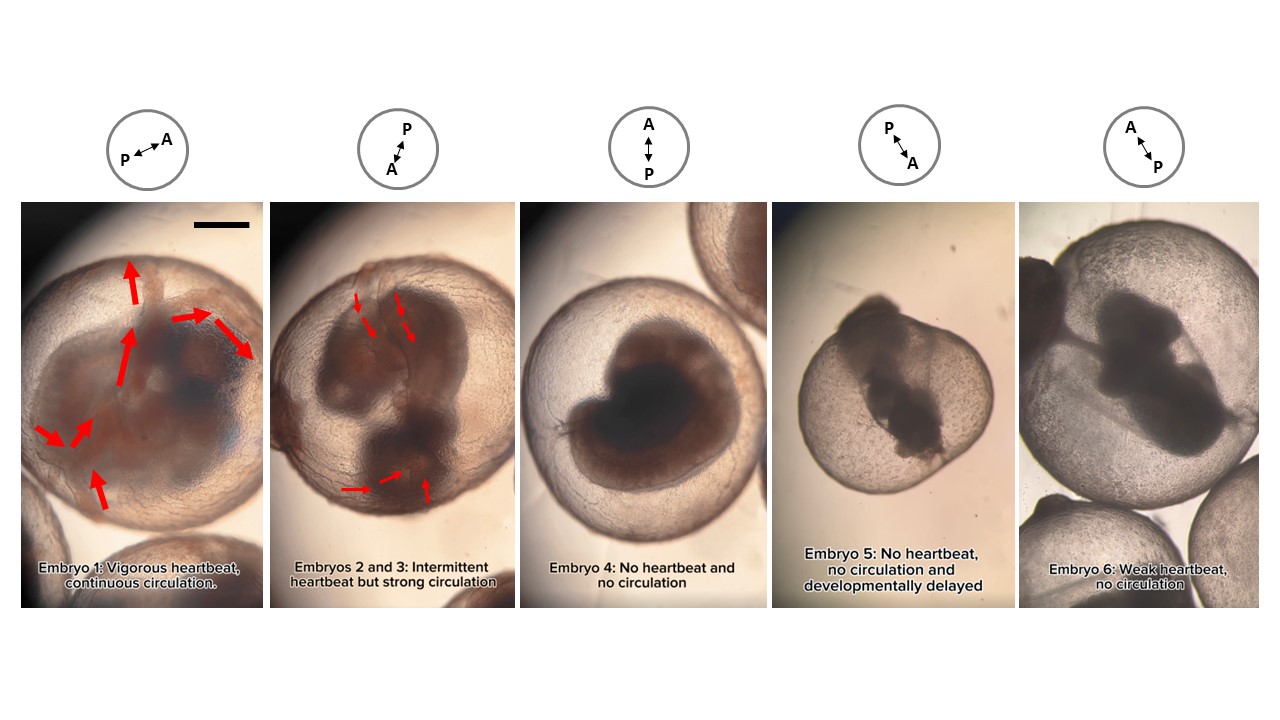

Supplement: Supplementary file 1 [file Image1.JPEG]
